# Atrial Fibrillation in Endurance Training Athletes: Scoping Review

**DOI:** 10.31083/j.rcm2406155

**Published:** 2023-05-26

**Authors:** Henrique M. Lobo, Ícaro G. Naves, Silvia Botelho Marçal, Camila Cassia Canzi, Amanda Braun Sabino Rodrigues, Antonio S. Menezes Jr

**Affiliations:** ^1^Medical and Life Sciences School, Pontifical Catholic University of Goiás, 74175-120 Goiânia, Goiás, Brazil; ^2^Internal Medicine Department, Medicine Faculty, Federal University of Goiás, 74690-900 Goiânia, Goiás, Brazil

**Keywords:** atrial fibrillation, cardiac remodeling, endurance exercise, high-performance athlete, physiopathology, sudden cardiac death

## Abstract

**Background:**

Moderate regular physical activity is indicated to avoid atrial fibrillation (AF), whereas athletes should be counseled that long-lasting vigorous sports engagement may cause AF, according to the 2016 European Society of Cardiology (ESC) recommendations for AF treatment. Exercise and AF are complex.

**Objectives:**

To evaluate the relationship between Endurance training and AF, in addition to the starting point/trigger by which Endurance Training causes impairment of cardiac function and AF, considering the time and intensity of Endurance training.

**Materials and Methods:**

We synthesized evidence from articles published in the PubMed, EMBASE, and SciELO databases using their respective Boolean operators. A total of 112 original articles related to AF and endurance athletes published up to the year 2023 were reviewed.

**Results:**

Our study verified multiples aspects of the genesis of AF in athletes, such as cardiac adaptations to exercise, disturbances in cardiac injury biomarkers, sex differences in cardiac adaptations and their role in AF risk, and the relationship between body composition (height, weight, and physical fitness) and AF pathogenesis.

**Conclusions:**

Variations in cardiac structure (increased atrial thickness and size in addition to myocardial fibrosis) and significant increases in vagal tone (sinus bradycardia and imbalances in sympathetic and parasympathetic activation) shorten the refractory period shortening in athletes, induce the onset of re-entrance mechanisms, and serve as ectopic triggers that can lead to AF.

## 1. Introduction 

Atrial fibrillation (AF) is a cardiac arrhythmia characterized by the disorganization of atrial electrical activity. It is the most common arrhythmia in the general population and may result in complications such as stroke, heart failure, myocardial infarction, peripheral arterial embolism, or death [1, 2]. However, AF is difficult to diagnose because it is often asymptomatic, and patients may experience nonspecific symptoms. AF is usually associated with older age [1, 2, 3, 4], being more common among individuals over 65 years of age and rarely occurring before the age of 25, which can be explained by age-related cardiac changes, such as a reduced number of cells in the electric impulse conduction system [2, 5]. The pathophysiology of AF is explained by the presence of several factors, including hemodynamic (increased intra-atrial pressure), structural (myocardial fibrosis), electrophysiological (refractory period shortening, myocardial conductivity alteration), modulatory (increased vagal tone), and triggering factors (ectopic loads especially of the pulmonary vein, extrasystoles, sinus bradycardia) [2, 6, 7, 8, 9, 10, 11, 12, 13]. Based on these pathophysiological mechanisms, physical activity has been cited as a possible risk factor for AF. Indeed, the adaptations and morphophysiological changes that occur due to physical exercise produce some of these factors, such as electrical and morphological remodeling of the myocardium [8, 14].

According to the 2020 European Society of Cardiology (ESC) Guidelines on sports cardiology and exercise in patients with cardiovascular disease, physical activity (PA) is defined as “any bodily movement produced by the skeletal muscle that results in energy expenditure”. Exercise or exercise training, on the other hand, is defined as “PA that is structured, repetitive, and purposeful to improve or maintain one or more components of physical fitness”. An athlete is defined as a “person whose main occupation is physical exercise, dedicating several hours of all or most days to the practice and improvement of one or more physical exercises” [15].

PA helps fight against several cardiovascular risk factors, including AF. Thus, regular physical activity is important for mitigating cardiovascular risks, especially those associated with obesity, metabolic syndrome, dyslipidemia, and hypertension [3, 6, 16]. Despite the demonstrated cardiovascular benefits of PA in several studies [3, 6, 17, 18], the relationships between intensity, exercise duration, and AF risk remain obscure. Athletes are required to maintain a certain level of effort for as long as possible. For example, the Copenhagen City Heart Study noted that male and female runners have a life expectancy of approximately 6 years longer than sedentary people; however, this increase in life expectancy was observed in groups that ran at low or moderate intensities and was not noted in individuals who engaged in higher-intensity running, which was typically defined as more than three weekly running sessions at greater intensity and for a longer duration (average: >4 hours/week) [3, 5, 6, 7, 17, 19, 20, 21, 22, 23].

Considering the increased participation in endurance sports in recent decades, there may be an increased risk of asymptomatic AF among athletes engaged in high-intensity forms of PA, such as the triathlon [3]. In addition, other endurance activities such as cycling, long-distance running, and cross-country skiing have been associated with increased AF risk [4]. Camm *et al*. [24] reported an estimated AF incidence of approximately 5–10% in athletes, reaching up to 10 times higher than in non-athletes of the same age. Thus, to evaluate if the risks of AF exceed the advantages of exercise and whether there is such a point as excessive amounts of a positive thing, this review aims to evaluate the current literature to answer the question: how may exercise raise the risk of AF?

## 2. Materials and Methods

Scoping reviews are an excellent technique for determining the scope or coverage of a body of literature on a certain issue, providing a clear indication of the volume of literature and studies available and an overview of its focus [25, 26, 27]. Scoping reviews are useful for investigating new information when it is unclear what other, more specific questions can be presented and valuable addressed by a more precise systematic review [27, 28]. They can report on the forms of evidence that address and inform field practice, as well as the methodology used in the research [27].

The overarching goal of scoping reviews is to identify and map the available evidence. So, some of the purposes for which scoping review may be useful are identifying the many sorts of evidence available in a specific field; clarification of major concepts or definitions in the literature; to investigate how research on a specific topic or field is carried out identifying important qualities or elements associated with a concept; as a precursor to a systematic review; identifying and analyzing knowledge gaps [27].

To accomplish so, the PRISMA extension for Scoping Reviews (PRISMA-ScR) was employed, which was created in accordance with published instructions from the EQUATOR (Enhancing the QUAlity and Transparency of Health Research) Network for the development of reporting criteria [28].

**Fig. 1.**
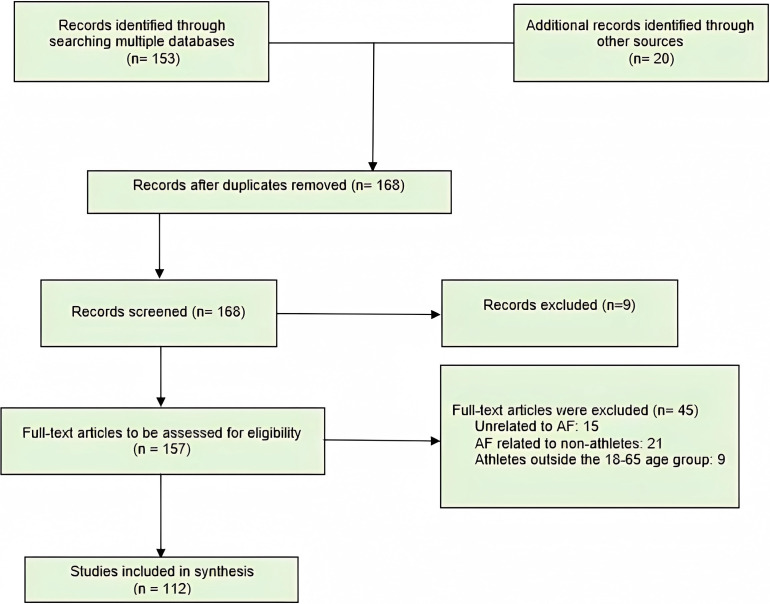
**Flowchart of selected studies (PRISMA-ScR)**. AF, Atrial Fibrillation.

A thorough survey of the published research was carried out utilizing the databases PubMed, EMBASE, and Scielo up to the year 2023. The review papers that were published on the topic as well as the reference lists of the publications that were retrieved, were also reviewed to look for qualifying manuscripts. The publications that had nothing to do with AF or endurance athletes were left out of the review, but every study that was published was chosen for inclusion. Based on this selection, 153 articles were obtained and organized into a folder in Zotero, stable release 6.0.18 (Corporation for Digital Scholarship; Vienna, Virginia, United States) for full reading; those articles that were excluded from the proposed discussion or that had significant methodological biases were excluded once more. As a result, after an exhaustive review of the articles that were chosen, a total of 112 pieces were included (Fig. 1), and 3 more studies were included at the suggestion of the reviewers.

Eligibility criteria: Articles with patients between 18–65 years old, without pulmonary, cardiovascular, or severe kidney disease, practitioners of endurance-type physical training. Papers were not filtered by language or time.

Ineligibility criteria: Articles with patients under 18 years old or over 65 years old, articles that included patients with severe cardiovascular, renal, and pulmonary comorbidities. Articles such as letters to the editor, viewpoints, and abstracts.

## 3. Results and Discussion 

**Fig. 2.**
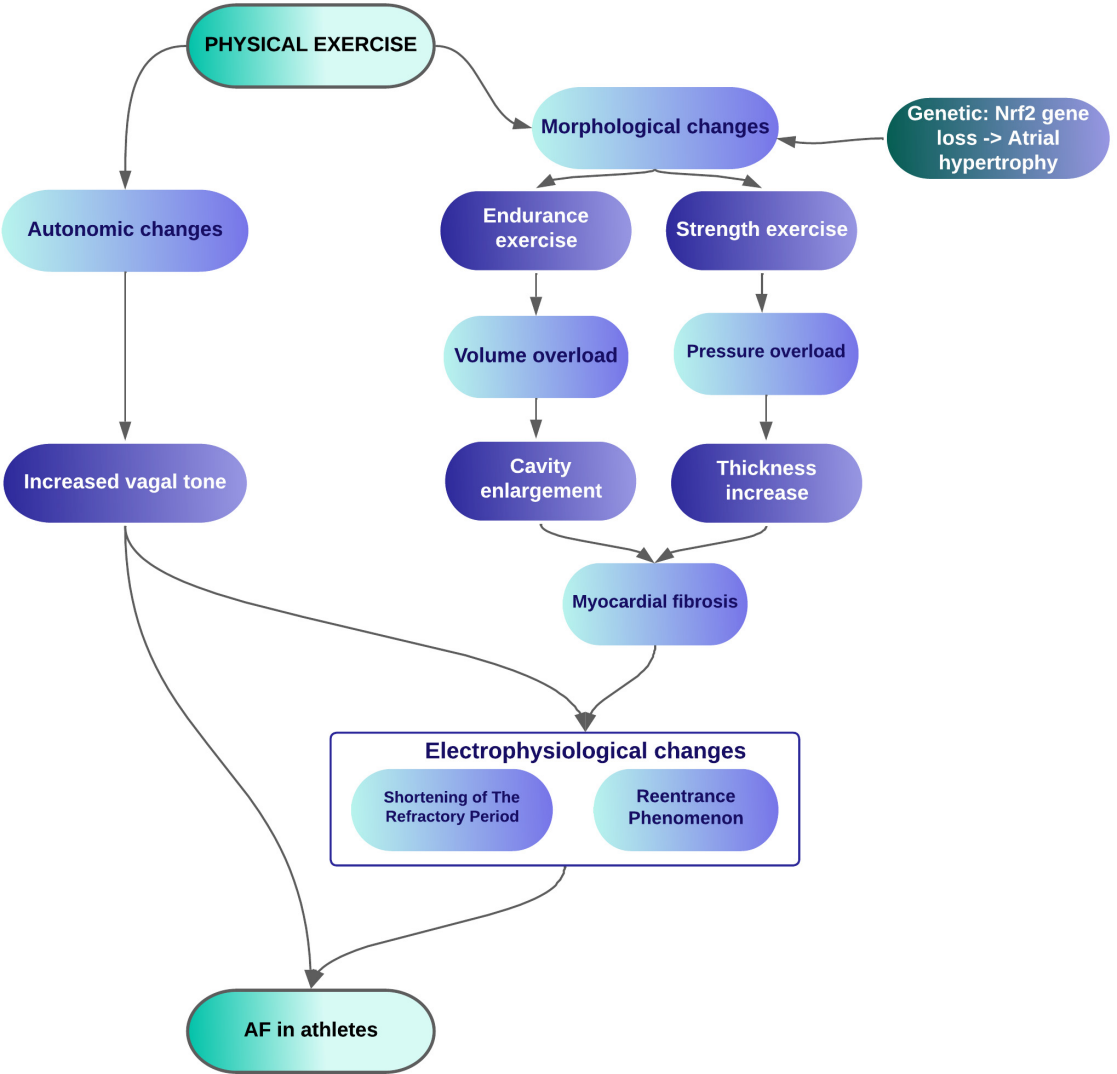
**Mechanisms triggering AF in response to physical exercise**. AF, atrial fibrillation.

The evidence obtained from the studies was based on the following categories: cardiac adaptations in response to physical exercise, markers of cardiac injury in athletes, differences in sex-related cardiac adaptation and their influence on AF risk, the relationship between physical fitness and AF, the role of height and weight in AF risk, exercise-induced atrial electrical remodeling, and AF in endurance athletes (Fig. 2).

### 3.1 Cardiac Adaptations in Response to Physical Exercise

The set of cardiac adaptations in response to physical exercise is known as the “athlete’s heart”, consisting of morphophysiological changes, in addition to presenting characteristic complications, the most serious being AF. Therefore, it’s essential to comprehend what the athlete’s heart is all about understanding the process of AF genesis in endurance athletes.

#### 3.1.1 The “Athlete’s Heart”

The athlete’s heart refers to cardiac adaptations to endurance training and may involve the expansion of all four cardiac chambers [8]. It is known that 4 or more hours per week of severe endurance exercises in a short period (2–4 months) can cause anatomical, electrical, and functional changes in the heart. This remodeling process responds to an overload state and is generally considered reversible and benign [14, 23, 29, 30].

#### 3.1.2 Long-Term Adaptations of the “Athlete’s Heart” and AF

In a large population-based sample, several measures of fitness and physical activity exhibited inverse relationships with future cardiovascular disease (CVD) events and all-cause mortality. Genetic risk for coronary heart disease (CHD) and AF was shown to be inversely associated with age, gender, and smoking status stepwise across all three risk categories [31]. The physiological demands of the heart increase sharply during endurance exercises. Removing the parasympathetic vagal tone and the initial reaction of the sympathetic nervous system to exercise results in an initial increase in heart rate. Thus, catecholamine release in the nerve terminals and subsequent “overflow” of epinephrine and norepinephrine in the systemic circulation are signs of sympathetic nervous system activation. These hormones also increase contractility and heart rate, increasing cardiac output and systolic volume. During the early stages of endurance exercise, these neurohormonal reactions increase cardiac output; however, prolonged endurance exercise can lead to a decline in cardiac function. For example, according to a meta-analysis that included 294 patients from 23 studies, after endurance exercise, there was a relative drop of 2% in the left ventricular ejection fraction (LVEF). These decreases in exercise-induced LVEF are most often observed in untrained people undergoing moderate-intensity (3 hours) exercises and in athletes training for ultra-endurance competitions (10.5 hours) [7, 8, 9, 32].

#### 3.1.3 Changes Caused by Endurance Training Versus Strength Training

**Fig. 3.**
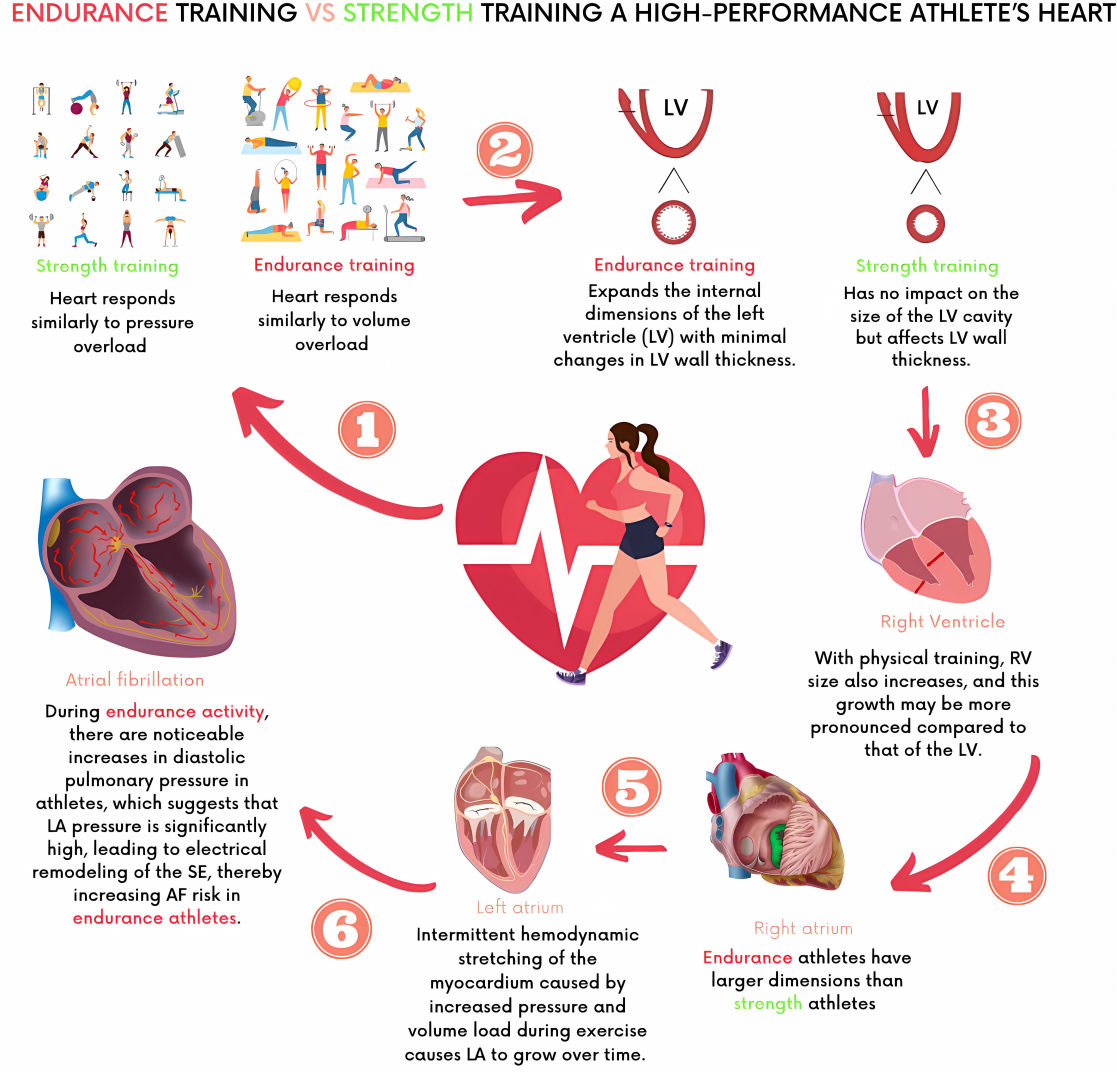
**Endurance training versus strength training**. LV, Left Ventricle; RV, Right Ventricle; LA, Left Atrium; AF, Atrial Fibrillation; SE, Septal Atrial Endocardium.

A high-performance athlete’s heart adapts to prolonged endurance and strength training in a manner similar to how a healthy person’s heart responds to volume and pressure overload, respectively. Thus, endurance training expands the internal dimensions of the left ventricle (LV) with minimal changes in LV wall thickness. In contrast, strength training does not impact the size of the LV cavity, but it affects LV wall thickness. An Italian study showed that endurance athletes had significantly larger dimensions of the left atrium (LA) and LV but did not have significantly thicker LV walls. In addition, previous studies showed that, compared with team sports, endurance sports present an increased risk of AF after controlling for accumulated hours of activity [8, 14, 32, 33, 34, 35, 36, 37, 38, 39, 40], as shown in Fig. 3.

Atrial reservoir function is regulated by ventricular systolic function. Studies have shown that the function of the right atrium (RA) reservoir is reduced in medium-distance runners and to a greater extent in long-distance runners, following the same pattern as the right ventricular (RV) function and confirming a dose–response relationship between exercise load and degradation in right-side cardiac performance [32, 39].

#### 3.1.4 Myocardial Fibrosis

Long-term or constant exercise can cause or accelerate the development of cardiac fibrosis. Eccentric ventricular hypertrophy, diastolic dysfunction, atrial dilation, and collagen deposition in the RV and both atria develop in rats forced to run for 16 weeks, equivalent to 10 years of physical endurance training in humans [5, 8, 9].

The myocardial extracellular matrix accumulates collagen, a sign of myocardial fibrosis. Myocardial fibrosis may have a non-ischemic origin, although it occurs more frequently after myocyte injury due to ischemia. In addition, myocardial fibrosis decreases ventricular compliance, which may result in atrial enlargement, AF, and heart failure with preserved ejection fraction [5, 38, 41, 42].

When the surface of a hypertrophic cardiomyocyte is greater than the distance over which oxygen can flow in its gradient from neighboring capillaries, the cell dies, leading to fibrosis and myocardial contractile depression [38, 43, 44]. Physical exercise can activate Akt, a serine/threonine protein responsible for cell proliferation in various cell types, and the Akt pathway may be involved in the pathological and healthy development of the heart. After 2 weeks of strenuous exercise in animal models, cardiac expression of the Akt pathway led to reversible hypertrophy; however, after 6 weeks of intense training, it led to irreversible cardiomyopathy with reduced capillary density and cardiac fibrosis. In addition, a previous study reported that patients with pathological hypertrophy and heart failure exhibited elevated angiotensin II (Ang II), catecholamine, and endothelin-1 (ET-1) levels compared to controls. Insulin-like growth factor 1 (IGF1) is released during postnatal development and physical training and is increased in swimming-trained and veteran athletes [38]. Thus, IGF1 induces healthy cardiac hypertrophy by activating the molecular PI3K-Akt pathway, while Ang II and ET-1 cause pathological cardiac hypertrophy by activating the mitogen-activated protein kinase (MAPK) and calcineurin pathways. Therefore, apoptosis and necrosis are linked to pathological hypertrophy. In this case, lost myocytes are replaced by excessive collagen deposits. Increased ventricle stiffness due to excessive collagen deposition results in impaired contraction and relaxation in addition to fibrosis of the electrical conduction system, which may cause AF [3, 5, 8, 10, 38, 44].

### 3.2 Markers of Cardiac Injury in Athletes 

**Fig. 4.**
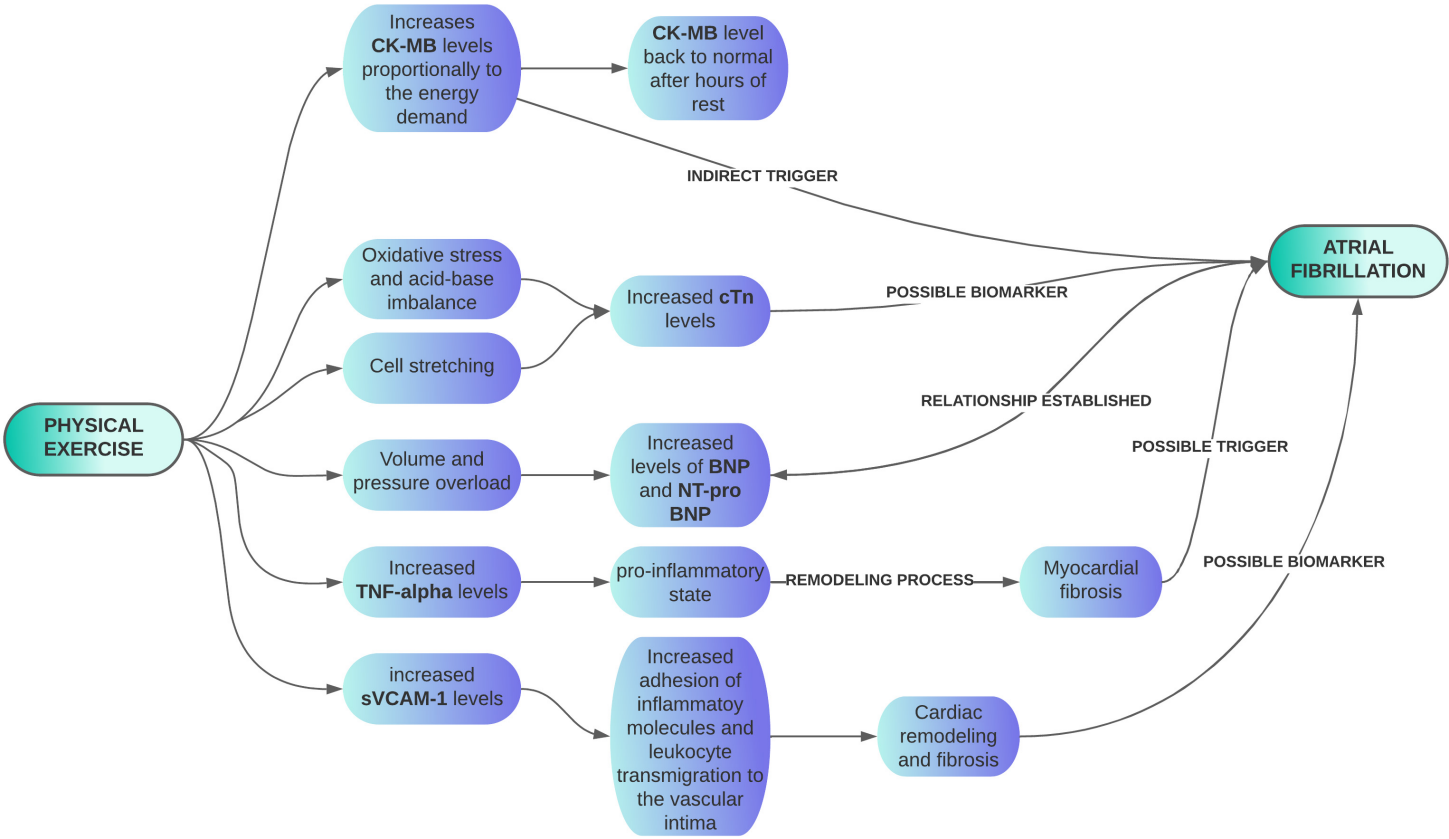
**Biomarkers alterations to physical exercise related to AF**. AF, Atrial Fibrillation; BNP, brain natriuretic peptide; CK-MB, Creatinofosfoquinase-MB; NT-proBNP, N-terminal-pro-BNP; TNF, tumor necrotizing factor; sVCAM-1, Soluble Vascular Cell Adhesion Molecule-1; cTn, cardiac troponins.

During extreme exercise, repeated cycles of oxidative stress and mechanical deformation of the heart muscle can damage the cardiomyocyte cell membrane, which explains the increase in levels of multiple cardiac injury biomarkers such as creatine kinase-myoglobin binding (CK-MB), cardiac troponins (cTn), and type B natriuretic peptide (BNP) [14, 22, 35]. Although biomarkers typically normalize a few days after intense exercise, researchers have speculated that repeated episodes of myocardial damage may precipitate pathological changes such as ventricular fibrosis. These fibrotic areas can constitute a proarrhythmic substrate, providing a slow conduction area and consequently increasing the probability of re-entry phenomena, thus enabling AF deflagration due to fibrosis caused indirectly by strenuous exercise [35], as seen in Table 1. Fig. 4 illustrates the relationship between physical exercise and biomarkers in the genesis of AF.

**Table 1.** ** Biomarkers related to cardiac remodeling and AF**.

Biomarker	Function	Alteration
CK-MB	Myocardial injury biomarker	Elevated in athletes, reaching up to 8% (Right Ventricle) versus 1% in the general population
Troponins (cTn)	Myocardial injury markers	Rise during exercise proportionately to cardiac energy demand during activity and return to normal levels hours after the end of the exercise
BNP and NT-proBNP	Markers of reduced ventricular function and heart failure; increased in patients with AF without structural problems	5–10× increase in levels after physical exercise in endurance athletes
TNF-α	Factor related to cell apoptosis and immune system signaling	Expression increased by physical exercise only in the atria, causing atrial myocardial fibrosis
TIMP-1, CITP, PICP, galectin-3, miR-21	Biomarkers of collagen synthesis and degradation	Elevated in older high-performance athletes
sVCAM-1	Biomarker of fibrosis and cardiac remodeling	Increased plasma levels in athletes
miR-1, miR-30, miR-133	Arrhythmogenic remodeling mediators	Increased levels in high-performance athletes

AF, atrial fibrillation; CK-MB, creatine kinase-myoglobin binding; BNP, Brain natriuretic peptide; NT-proBNP, N-terminal fragment of pro-brain natriuretic peptide; TNF-α, Tumor necrosis factor alfa; TIMP-1, Tissue inhibitor of metalloproteinases-1; CITP, C-terminal telopeptide of collagen type 1; PICP, carboxyterminal propeptide of collagen type I; miR, microRNA; sVACM, Soluble Vascular Cell Adhesion Molecule-1.

#### 3.2.1 CK-MB

Although CK-MB typically accounts for approximately 1% of the total skeletal muscle tissue CK, it can represent up to 8% of that in endurance athletes. In addition, increased CK-MB concentrations in endurance athletes are not a component factor, but rather an adaptation to training, as demonstrated by the fact that muscle CK-MB concentration increases with physical training [8, 14, 17, 35].

#### 3.2.2 Troponins

Younger age, the existence of cardiovascular risk factors, inexperience in the running, longer duration and intensity of exercise, and increased dehydration with exercise contribute to higher increases in exercise-induced cTn [8, 14, 35]. Exercise intensity is the most powerful predictor of cTn release, followed by younger age and longer activity duration among skilled marathoners. This finding indicates that cardiac exercise work and cTn response to exercise are closely correlated [8, 22, 35]. Exercise may increase cardiac sarcolemma permeability due to mechanical stress in cardiomyocytes, increased generation of oxidative radicals, alteration in acid-base balance, and passive transport of cTn from the intracellular compartment to the extracellular compartment. Thus, the cardiac plasma membrane may be temporarily ruptured as a result of cell stretching, followed by cTn release, and given the intensity of exercise, higher cTn levels are more frequent during triathlons or cycling [38]. Accordingly, cardiac demand during exercise is mainly influenced by intensity [8, 23, 38, 45]. The difference between cTn levels increase by infarction, and physical exercise is that the cTn increased by physical exercise returns to normal levels in less time than infarction-increased cTn levels [46, 47].

Finally, studies elucidating the possible relationship between AF and troponin elevation during exercise are still lacking.

#### 3.2.3 BNP and N-Terminal-Pro-BNP (NT-proBNP)

The serum BNP level is a well-known measure of increased myocardial strain and a clinical predictor of worsening heart failure. It is also higher in patients with AF who do not have structural heart problems. It increases with high AF load and decreases with cardioversion or catheter ablation [22, 45, 46, 47, 48, 49]. At rest, the BNP and NT-proBNP levels of endurance athletes are comparable to those of people not trained at the same age but increase 5 to 10 times after exercise in those who participate in endurance exercise events [8, 38, 45].

#### 3.2.4 Tumor Necrosis Factor Alpha (TNF-α)

High-intensity physical exercise exclusively increases the activation of NFκB, a protein complex transcribed by TNF-α, and p38 MAPK, a class of stress-sensitive kinases related to immunological activation, cell cycle control, and other signaling pathways. This activation plays a role in cardiac remodeling and promotes myocardial fibrosis, consequently constituting a triggering factor for AF in athletes [50].

#### 3.2.5 Other Plasma Markers

Compared with age-matched sedentary controls, elite endurance athletes aged 45–75 years with 10 years of competitive experience and currently running 30 miles/week exhibit increased plasma markers of collagen synthesis and degradation, including metalloproteinase matrix type I tissue inhibitor (TIMP-1), carboxyterminal collagen telopeptide type I (CITP), Procollagen type I carboxy-terminal propeptide (PICP) , galectin-3, and various circulating profibrotic microRNAs, especially miR-21 [3, 5, 7, 10, 35]. Athletes with the highest TIMP-1 levels exhibit LV hypertrophy. Experienced endurance athletes may have cardiac fibrosis based on biochemical evidence of aberrant collagen renewal, and fibrosis can induce AF by slowing conduction [3, 5, 7, 8, 10, 51].

Another potential biomarker of fibrosis and cardiac remodeling caused by exercise is soluble vascular cell adhesion molecule-1 (sVCAM-1), which is essential for the adhesion of inflammatory molecules and leukocyte transmigration to the vascular intima. A study showed that Caucasian male runners engaged in high-intensity exercise have increased plasma levels of sVCAM-1. Thus, sVCAM-1 is a possible biomarker for evaluating and monitoring potential negative effects, including AF, on LA structure and function in high-performance athletes because sVCAM-1 level is positively linked to the increased LA volume, as shown in Fig. 4 [37, 52].

### 3.3 Sex-Related Differences in Cardiac Adaptation and Their Influence on AF Risk

Female athletes are less likely to present with thicker LV walls and smaller LV and LA diameters [8, 53, 54]. Absolute atrial volumes are higher in men than in women. In addition, men have higher volumes of LA related to height and body surface area than women, and the same is true for systolic volume indices [53, 54, 55].

Higher systolic blood pressure and androgenic hormones are underlying factors that may explain why the atria are larger in male athletes. One study reporting higher systolic blood pressure in male athletes than female athletes suggested that this difference can impact atrial remodeling [53]. Androgenic hormones that affect cardiac protein synthesis may partly contribute to a larger atrium. In addition, cardiovascular adaptations resulting from exercise may be influenced by skeletal muscle mass, training volume, and plasma volume expansion. In addition, previous studies indicated that women had smaller atria, lower LV mass and wall thickness, and different autonomic tones than high-intensity male athletes [44, 53, 54, 55, 56].

However, due to the scarcity of information on AF risk in female endurance athletes, the role of sex is not fully understood. The Tromsø2 Study in Norway, which followed 10,184 women for 20 years, included many female participants [7]. It revealed a U-shaped curve similar to that in men when AF risk and cumulative exercise were correlated. Nevertheless, the risk of AF in female endurance athletes was similar to that in sedentary women [1, 7].

Unfortunately, comparative studies on the risk of developing AF in men and women are still scarce, despite the existence of relevant clinical cohorts in England. For instance, The Million Women Study has had over 100 publications since its inception in 1996 and is ongoing. In Norway, the Tromsø Study, which began in 1974, also released hundreds of publications during its seven-stage course, which was completed in 2016. Its eighth stage, called Tromsø8, is scheduled for completion by 2025.

### 3.4 Relationship between Physical Fitness and AF

Any organized and structured intervention aimed at improving or maintaining cardiorespiratory fitness (CRF) or health, achieving sporting goals, or both is called physical training [57]. Physical fitness should not be confused with habitual PA, even if PA habits are the main predictor of physical fitness. Physical fitness can be easily assessed using an exercise tolerance test, and PA and fitness can be separate physiological indicators of cardiovascular disease [57, 58, 59].

In young or middle-aged athletes without cardiac structural abnormalities, sustained endurance exercise is associated with a 3 to 10 times higher risk of AF, which is not observed in non-athletes. According to O’Keefe *et al*. [23], individuals with an exercise capacity of less than 6 metabolic equivalents (“METs”, which is a unit of measurement used to quantify the metabolic demand of an activity to the basal demand for the individual to remain at rest, being used to assess the volume of activity) have higher rates of AF than individuals who are more physically fit [58, 59, 60, 61, 62, 63, 64, 65]. Even small amounts of exercise, starting with 5 MET hours/week, seem to decrease AF risk, with the greatest benefits shown at 20 MET hours/week (approximately 2 hours and 45 minutes/week). A recent UK Biobank cohort survey (n = 402,406) demonstrated that getting more than 500 MET minutes/week was associated with a lower incidence of AF. The World Health Organization’s PA guidelines define 150 minutes of moderate-intensity PA or 75 minutes of vigorous-intensity PA as equivalent to at least 450 MET minutes/week, effective for cardiovascular protection against various diseases, especially AF. In fact, exceeding existing PA patterns between 500 and 1500 MET minutes/week was associated with a 5–10% and 6–20% decrease in AF incidence in men and women, respectively. Thus, the risk of AF recurrence was 13% lower for each increase in MET in initial CRF. As such, the probability of a recurrence can be predicted using initial fitness levels [66, 67, 68, 69].

**Fig. 5.**
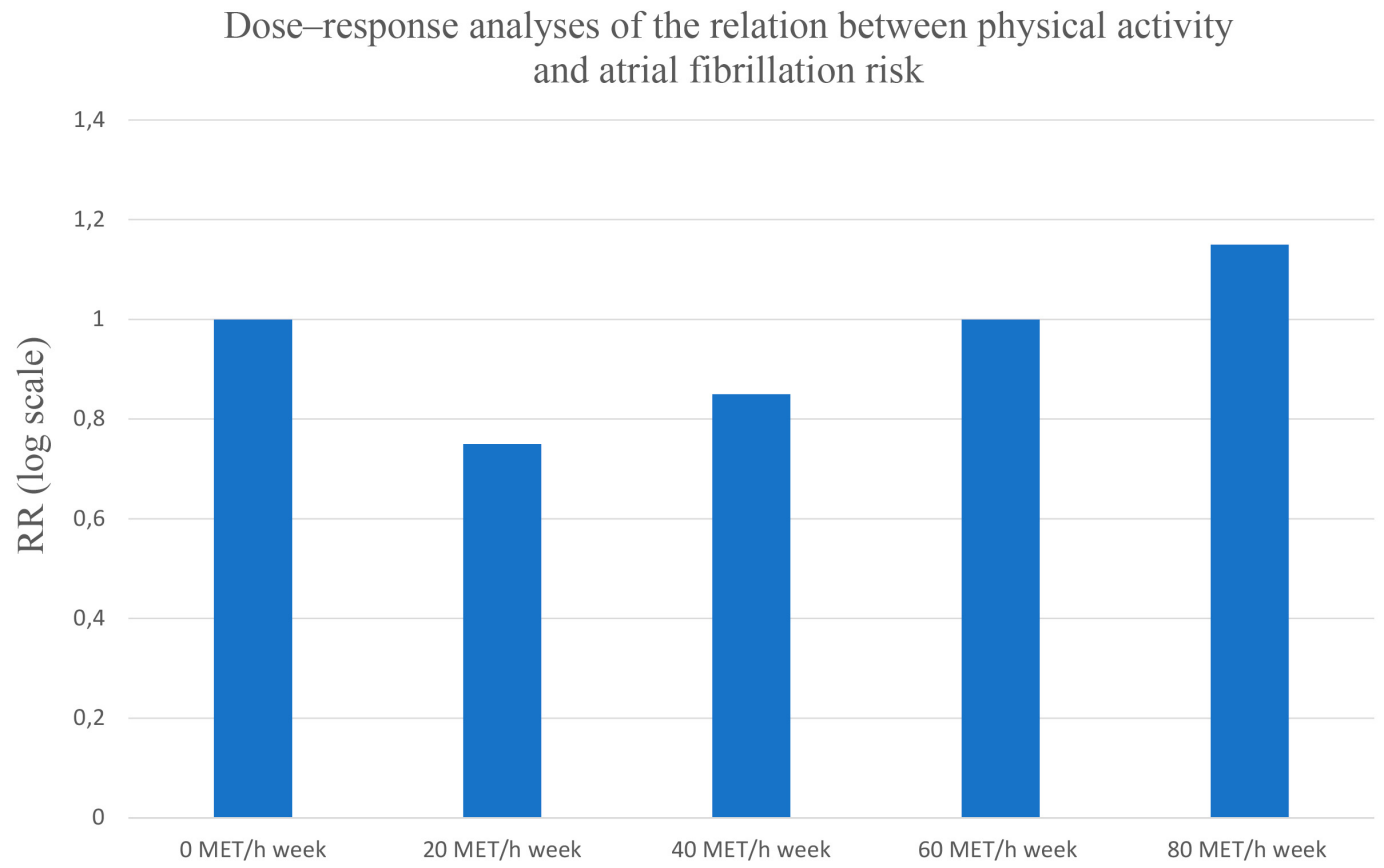
**Dose–response analyses of the relation between physical activity and atrial fibrillation risk**. RR, risk rate. *Based on: PA level has a U-shaped relationship with AF risk, with active groups having a 12% lower risk than sedentary groups [70].*

PA level exhibits a U-shaped relationship with AF risk, as shown in Fig. 5 (Ref. [70]). In previous research, the active group (500–1000 MET minutes/week) had a 12% lower AF risk (adjusted risk rate [RR]: 0.88, 95% confidence interval [CI]: 0.80–0.97) than the sedentary group. However, insufficiently active (1–500 MET minutes/week; HR: 0.94, 95% CI: 0.86–1.03) and extremely active (1001 MET minutes/week; HR: 0.93, 95% CI: 0.85–1.03) groups had a 6% and 7% decrease in AF incidence, respectively [70]. Moreover, improving physical fitness during the intervention was associated with a lower risk of AF recurrence. However, the risk of developing AF exceeded that of the sedentary group by 55 MET hours/week, or approximately 10 hours of intense exercise per week, which supports the U-shaped relationship between physical fitness and AF [69, 70]. Participation in endurance sports increased the risk of AF by two to ten times, and the number of accumulated hours of vigorous endurance training throughout life (specifically more than 2000 hours) was the most powerful predictor of exercise-induced AF [66]. In addition, a lower exercise capacity was independently associated with a higher CHA2DS2-VASc score, typically used for thromboembolic risk stratification in patients with AF [57]. The CHA2DS2-VASc score can predict exercise intolerance, particularly in male patients who are relatively young and middle-aged and have asthma-related AF [71, 72].

In exercise, volume and intensity should be considered since AF risk increases with increasing volume. When endurance exercise is performed more frequently (i.e., >4 times/week) and longer (i.e., >5 h/week), or when an accumulated amount of vigorous exercise >2000 hours is completed, a higher AF incidence is observed [23, 29, 30].

Finally, Franklin *et al*. [57] observed that individuals with AF who improved their fitness (up to 6 METS) during a physical training program showed a substantial reduction in AF load and symptom severity compared with those who did not improve and among those who were randomly assigned to interval aerobic training (>6 METS). Therefore, AF risk was higher in athletes than in non-athletes, who did not reach values >6 METS; however, these data indicate that people who are more physically able have the lowest risk [55]. A similar, significant relationship was observed between AF and CRF in previous studies involving young Swedes serving in the military [8, 31, 55, 66].

### 3.5 Height and Weight in AF Risk

Crump *et al*. [73] studied the relationship among height, weight, and physical fitness with AF in a cohort of 1,547,478 participants. After adjusting for all variables, they observed increased AF incidence with increasing height, weight, and physical fitness levels. Considering the importance of body structure in the athletic performance of an individual, weight, and height increase AF risk, especially when related to physical exercise [73].

### 3.6 Exercise-Induced Atrial Electrical Remodeling 

Atrial remodeling (i.e., dilation and atrial fibrosis) contributes to the pro-arrhythmogenic effects of high-intensity exercise. Therefore, atrial dilation is considered a physiological aspect of the heart’s adaptation to exercise; however, it also increases the vital myocardial mass needed to develop the fundamental processes of AF [5, 74].

In the atrium, fibrosis of the extracellular matrix obstructs regular electrical conduction, inducing heterogeneous electrical conduction and re-entry production, which has been observed in an exercise-induced animal model of AF; thus, fibrosis is considered a structural change inherent in AF [5].

In addition, numerous recent studies have suggested that oxidative stress is connected to pathways that stimulate atrial structural and electrical remodeling, resulting in atrial ectopy and interstitial fibrosis. The onset of AF is often triggered by delayed post-depolarizations, owing to an increase in the release of ${\mathrm{Ca}}^{2}$^+^ from the endoplasmic reticulum (ER) of cardiomyocytes [44, 55, 75, 76, 77]. *In vitro* exposure of primary cardiomyocytes to high glucose concentrations increases levels of ER stress and ${\mathrm{Ca}}^{2}$^+^ [2, 3, 75]. ER stress is a pathological state that can be triggered by a variety of cellular settings and events, including those occurring due to exercise (*e.g.*, excessive protein synthesis, impaired autophagy, energy deprivation, deficiency or nutritional excess, unregulated ${\mathrm{Ca}}^{2}$^+^ levels or redox balance, inflammation, and hypoxia) [2, 75].

### 3.7 AF in Endurance Athletes

Any disease that increases the size or pressure of the LA (*e.g.*, hypertension, left systolic or diastolic heart failure, and mitral valve stenosis or regurgitation) is a risk factor for AF. The probability of AF also increases with increased sympathetic and parasympathetic tone. The atrial refractory time is shortened by increasing the parasympathetic tone by reducing the inlet current of L-type calcium channels. In addition, atrial re-entry is facilitated by a shorter atrial refractory time, reducing the excitation wavelength [3, 8, 10, 19, 21, 43, 44].

The ability of exercise to reduce AF risk in athletes exhibits a U-shaped dose-response pattern, meaning that it is relatively less effective in high-intensity endurance athletes [7, 8, 11, 23, 57, 66, 71, 78]. Moderate activity levels are linked to a lower AF prevalence, probably by decreasing the risk of diseases such as hypertension and metabolic disorders, which can cause AF. In a cardiovascular health study, mild-to-moderate activity was associated with decreased relative risk of recent-onset AF. In contrast, sustained high-intensity exercise seems to increase the risk of AF [8, 72]. Data from nine studies involving 8901 people were reviewed to determine whether AF risk was higher in athletes than in the general population. The results indicated that, compared with the general population, athletes had a considerably higher chance of developing AF [79]. The number of days per week of intense physical exercise increased the AF incidence among healthy participants; even among athletes, AF risk seems to increase with the time and intensity of endurance exercise [8].

Although the exact mechanism underlying the development of AF in endurance athletes is unknown, it is likely a combination of an elevated parasympathetic tone and SE enlargement, especially in senior endurance athletes. Current knowledge of AF pathogenesis requires an ectopic trigger that causes inadequate depolarization and a susceptible fibrillogenic substrate or re-entrant mechanism that propagates the trigger. Evidence suggests that the autonomic nervous system plays a role in the initiation and maintenance of AF, contributing to both focal and re-entry processes. Vagal stimulation of the atrial myocardium can decrease the refractory period of atrial tissue and create atrial ectopic activity, leading to tortuous pathways and supraventricular tachyarrhythmias. Exercise can also promote AF by stimulating the sympathetic nervous system. Although vigorous exercise may be sufficient to cause this, additional sympathetic mimetic substances often worsen the situation [3, 7, 8, 10, 19, 33, 38, 44, 69, 71, 80, 81].

Premature atrial contractions, which may trigger AF, have been observed more frequently in athletes with many cumulative training hours. If an arrhythmogenic atrial substrate is present, this trigger may initiate an episode of persistent AF. Dilation and atrial fibrosis, which predispose patients to atrial re-entry, are characteristic of the atrial substrate of AF, while an increased vagal tone shortens atrial refractory time, which may facilitate re-entry and perhaps perpetuate AF. The length of the P-wave on electrocardiography correlates with atrial fibrosis, which can be demonstrated by surgical samples; however, it is not related to the atrial increase itself, both of which may be influenced by the practice of physical exercise [5, 7, 8, 10, 19, 33]. Furthermore, increased atrial pressure as measured via echocardiography may play a role in the etiology of AF related to physical exercise. A similar left atrial adaptation has been observed in marathoners, along with an elevated parasympathetic tone and atrial ectopic activity [7, 10].

The vagal characteristics of AF remain unclear. However, some criteria have been used in experimental studies to designate vagal AF, which include atrioventricular block, presence of asystole phases, sinus bradycardia, and increased heart rate variability (N50% in research). These have been observed in athletes with an extremely low resting heart rate, reaching more than 1s during asystole [82, 83, 84]. This study classified the vagal triggers for AF as follows: postprandial AF, nighttime AF, and AF without adrenergic triggers (exercise, emotion, and presence mainly during the day), even though many doctors do not seek triggers for AF (Fig. 2). Exposure to these triggers can lead to an imbalance in parasympathetic and sympathetic activation, ectopy, and changes in the atrial substrate, predisposing the individual to AF [85]. The vagal characteristics were postulated with the objective of elucidating the higher occurrence of paroxysmal AF in young athletes [86], being up to five times more common in athletes than in the general population, probably due to vagal hyperactivity [87].

Endurance athletes’ increased AF has been explained by many possibilities, but further study is required. Atrial electrical and mechanical remodeling may cause AF. Brugger *et al*. [88] divided male athletes into low, middle, and high-intensity training. High-intensity exercise increased LA dilatation and P wave duration, which are connected to AF. Echocardiographic left atrial wall strain was also enhanced, indicating greater atrial stretch during vigorous exercise. Marathon runners had enhanced parasympathetic tone and atrial ectopic activity, and left atrial adaptation [89]. The same group found that marathon runners had higher pro-atrial natriuretic peptides (pro-ANP) [90]. Atrial stretch releases pro-ANP. The mechanism connecting them needs additional investigation.

Paroxysmal AF is usually caused by pulmonary vein ectopy [91]. Wilhelm* et al*. [90, 92] found premature atrial beats increased with marathons and training hours in middle-aged non-elite runners. Former elite cyclists did not vary from age-matched golfers in early atrial beats [93].

Claessen *et al*. [94] also found considerable increases in diastolic pulmonary pressures during endurance exercise, indicating high left atrial pressures. Highly trained athletes may have atrial enlargement due to higher left atrial pressures during endurance exercise [90, 92, 95]. In certain persons, prolonged exercise stress may cause inflammation and fibrosis, which can cause arrhythmias [96, 97]. Left atrial cavity function cannot be determined from left atrial dimensions and volume alone. Brugger *et al*. [88] found that left atrial structural and electrical remodeling is not linked to atrial function in 95 amateur male runners over 30. However, 2D strain echocardiography significantly links diminished atrial function to paroxysmal AF [98].

Benito *et al*. [99] found atrial fibrosis in male Wistar rats. Significantly, stopping exercise reversed fibrotic alterations. Humans have not reversed fibrosis. Lindsay *et al*. [100] found pro-fibrotic markers in 45 top veteran athletes. These athletes had greater levels of three cardiac fibrosis biomarkers: PICP, CITP, and tissue inhibitor of matrix metalloproteinase type I (TIMP-1). Endurance exercise causes fibrosis. D’Ascenzi *et al*. [101] used novel echocardiographic methods to estimate myocardial stiffness, which directly relates to left atrial fibrosis. Athletes’ left and right atriums were normal or lower than normal compared to inactive people and showed no reaction to exercise [101].

Exertion promotes atrial remodeling and AF propagation through inflammatory cytokines. Pro-inflammatory cytokines, highly sensitive C-reactive protein (CRP), and leukocytes are higher in Swiss mountain marathon runners, as is signal averaged P wavelength, a measure of atrial conduction delay [102].

### 3.8 Future Prospects

#### 3.8.1 Genetics

Oxidative cellular alterations and redox imbalances in the atrium may be closely related to AF. In stressful situations, such as intense exercise, cardiomyocyte-produced reactive oxygen species can promote inflammation and activate downstream molecular pathways, which supports morphological and electrical models. Recent research indicates that loss of nuclear factor erythroid 2-related factor 2 (Nrf2), an antioxidant gene in the atria, may be linked to atrial hypertrophy and AF, indicating that maintaining the redox state is crucial for atrial health [3, 38, 44]. Moreover, a history of arrhythmias has been observed in approximately 5% of patients with AF and 15% of those with isolated AF who are referred for arrhythmia evaluation. In families, individuals, and several populations, some genes and loci related to AF and its substrate have been confirmed; however, some genes related to the development and risk of AF remain to be identified. When AF is caused by hereditary cardiomyopathies, it is classified as monogenic; when it is caused by common genetic variations linked to the early onset of AF in the general population, it is classified as polygenic [36].

#### 3.8.2 AF and Ang II

Ang II, a critical component of the renin-angiotensin system, activates several intracellular signaling pathways and increases cardiac cell proliferation and extracellular matrix protein synthesis in cardiac fibroblasts, resulting in cardiac remodeling [103]. Clinical trials have shown that inhibition of the renin-angiotensin pathway may prevent AF development or recurrence. The inotropic and chronotropic effects of Ang II on the heart have been documented, probably owing to the direct influence of Ang II on myocardial ionic channels [44, 103]. Finally, Ang II increases the myocardial automaticity of the pulmonary vein by activating the IP3 receptor and improving the ${\mathrm{Na}}^{+}$-${\mathrm{Ca}}^{2+}$ exchanger [103, 104].

#### 3.8.3 Treatment of AF in High-Performance Athletes

Research on effective treatment methods for AF in athletes is limited. In a case report, Cervellin *et al*. [105] described the efficacy of reducing the burden of physical exercise as a treatment for paroxysmal AF in a 32-year-old athlete, achieving complete improvement in symptoms and preventing the occurrence of new AF episodes. However, considering that the objective of AF treatment in athletes is to preserve athletic capacity since most of these high-performance athletes work competitively, exercise load reduction is infeasible, and more appropriate interventions are necessary [105, 106, 107, 108, 109]. Indeed, vitamin K antagonists, which are commonly used for AF treatment, are contraindicated for athletes given their detrimental impact on the athletic performance given the need for frequent blood tests, a large number of drug and dietary interactions, and a greater predisposition to hematomas and bleeding [110, 111]. Considering these limitations, a cohort study by Mandsager *et al*. [107] concluded that AF treatment through pulmonary vein isolation was equally effective between athletes and non-athletes, with no significant differences in AF recurrence and better preservation of athletic capacity in both groups.

#### 3.8.4 Screening of Athletes for AF

The loss of exercise capacity during AF is in the range of 15% to 20%, which demonstrates an urgent need to define sensitive and specific methods for early detection and screening of AF in athletes, focusing on preserving their athletic ability. In 2018, the U.S. Preventive Services Task Force concluded that there is insufficient evidence to support regular electrocardiogram screening for AF in asymptomatic individuals over 65 years of age [112]. MicroRNAs are essential mediators of pro-arrhythmogenic remodeling and can potentially be explored as biomarkers for cardiovascular diseases and sports-induced cardiac adaptations. However, these findings should be viewed cautiously, as a direct causal relationship between circulating levels of miRNAs in the blood and the development of AF remains to be established [21, 113]. Nonetheless, despite the absence of a clear relationship between these possible biomarkers and AF in athletes, it should be noted that “elite” runners exhibited higher miR-1, miR-30, and miR-133 levels than other runners in the Marathon Study, which were correlated with greater left atrial volumes [114, 115].

## 4. Conclusions

The available evidence indicates that the practice of endurance exercise exhibits a dose-response relationship with the risk of AF, which is influenced by exaggerated time and intensity of practice. This relationship is due to morphological and electrophysiological cardiac changes resulting from exercise, which provide a substrate for AF emergence and cause ectopic triggering. The stress on the cardiac chambers produced by intense exercise induces pathological hypertrophy, cardiomyocyte apoptosis, and excessive collagen deposition in the cardiac tissue, leading to myocardial fibrosis and triggering electrical remodeling, especially in the atria. This provides a mechanism for re-entry, which is responsible for AF onset in most cases.

Finally, the relationships between markers of injury and cardiac fibrosis in athletes and the response of each marker to endurance exercise have not been sufficiently elucidated to establish the parameters for AF screening and diagnosis in high-performance athletes. However, recent discoveries regarding the influence of sVCAM-1 on the dilation and electrical remodeling of the LA and the roles of markers such as TIMP-1, CITP, PICP, galectin-3, and several profibrotic microRNAs (*e.g.*, miR-21) may help to improve evaluation and monitoring of the potential negative effects of high-intensity training on the heart in athletes. These data may also aid in identifying the mechanisms that trigger AF in the general population.

## Data Availability

The datasets used and/or analyzed during the current study are available from the corresponding author on reasonable request.

## Supplementary Material

Supplementary material associated with this article can be found, in the online version, at https://doi.org/10.31083/j.rcm2406155.
